# The Impact of COVID-19 Interventions on Influenza and Mycobacterium Tuberculosis Infection

**DOI:** 10.3389/fpubh.2021.672568

**Published:** 2021-05-21

**Authors:** Yiman Geng, Gang Li, Leiliang Zhang

**Affiliations:** ^1^Department of Clinical Laboratory, Henan Provincial People’s Hospital, Peolple’s Hospital of Zhengzhou University, Zhengzhou, China; ^2^Institute of Basic Medicine, The First Affiliated Hospital of Shandong First Medical University, Shandong, China; ^3^Science and Technology Innovation Center, Shandong First Medical University & Shandong Academy of Medical Sciences, Shandong, China

**Keywords:** COVID-19, SARS-CoV-2, influenza, Mycobacterium tuberculosis, non-pharmaceutical interventions

## Abstract

A series of public health interventions have been implemented to prevent the transmission of SARS-CoV-2 in China. However, the effect of non-pharmaceutical interventions to COVID-19 on the incidence of the influenza virus and Mycobacterium tuberculosis infections is not clear. In current study, we analyzed surveillance data on influenza and Mycobacterium tuberculosis from Henan Provincial People’s Hospital in Zhengzhou, Henan province, China from 2019 to 2020. The monthly positive test rate for influenza and Mycobacterium tuberculosis to estimate transmissibility changes was calculated. The positive detection rate of influenza A declined significantly during the implementation of inventions in 2020, from a total positive rate of 17.69% in 2019 to 5.77% in 2020. Similarly, a 2.15% reduction in positive detective rate was seen for influenza B, from a total positive rate of 5.15% in 2019 to 3% in 2020. The positive rate curve of Mycobacterium tuberculosis measured by x-pert in 2020 remained above the curve in 2019 from March to June, and August, showing the rising trend under these precautions. Our study suggests that non-pharmaceutical public health interventions likely reduced influenza transmission significantly and have less effect on Mycobacterium tuberculosis transmission in 2020.

## Introduction

Coronavirus disease 2019(COVID-19) caused by severe acute respiratory syndrome coronavirus 2 (SARS-CoV-2) was first reported in China in late December 2019 and has spread to become a global pandemic, raising worldwide public concern (1). During a public health crisis of global proportions caused by the pandemic, to control the rapid transmission of this coronavirus disease in 2019, the Chinese government has implemented various non-pharmaceutical public health interventions including quarantine, testing of all coming travelers or returnees, mask-wearing, rapid contact tracing, massive reverse-transcription polymerase chain reaction (RT-PCR) testing for case detection, personal hygiene and social distancing measures (2, 3). With unprecedented public health interventions, local transmission of this epidemic has been remarkably contained in China (4). However, the efficacy of the community-wide masking of the population on the other respiratory pathogens such as influenza virus and Mycobacterium tuberculosis (MTB) remains inconclusive.

Influenza A and influenza B are the predominant viral members of seasonal influenza due to the principle of dominance by competitive circulation (5). Meanwhile, MTB is a cause and significant pathogens of tuberculosis, with the respiratory tract as the main transmission route (6). In the Henan province of China, the general population practiced protective infection control measures and interventions at an early stage of the local COVID-19 epidemic. Here, we described the comparative epidemiology of influenza and MTB during the 2019 and 2020 in Henan Provincial People’s Hospital to investigate whether the non-pharmaceutical interventions affect the transmission of the two respiratory pathogens. In the future, these non-pharmaceutical public health interventions would be very meaningful for future respiratory illness controlling.

## Methods

### Laboratory Diagnosis of Influenza A and B Virus

In our hospital, GeneXpert system that detects tuberculosis was launched in August 2016 and the start time of the test item of antigen detection for influenza A and B viruses was from October 2018. We extract data for outcomes from the laboratory information management system in hospital for retrospective study. After ethical approval was given by the ethical committee of Henan Provincial People’s Hospital (registration number 20090020), patient-related data (test items and results of test) were collected from their medical files retrospectively. The specimens of throat-swabs were collected by clinicians and sent to the clinical detection and diagnosis laboratory of Henan Provincial People’s Hospital to test for influenza A and influenza B virus antigen using a colloidal gold immunochromatography assay (Rapid influenza virus antigen test kits, Guangzhou Wondfo Biotechnology Co., Ltd, China).

### Laboratory Diagnosis of Mycobacterium Tuberculosis

The clinical specimens for detecting MTB include sputum, bronchoalveolar lavage, pleuroperitoneal effusion, and cerebrospinal fluid. We applied a rapid molecular diagnostic assay to detect MTB in sputum and other specimens using the x-pert MTB/RIF automated nucleic acid amplification test (x-pert MTB/RIF; Cepheid, Sunnyvale, CA, USA).

### Statistical Analysis

We compared incidence rates between groups using Pearson’s chi-square test or Fisher’s exact test. All statistical analyses were performed using Prism 6.0 (GraphPad Software, La Jolla, CA). Two-sided *p*-values < 0.05 were considered statistically significant.

## Results

### Epidemiology of Influenza Virus in Patients From Henan Provincial People’s Hospital

**Figure 1:**
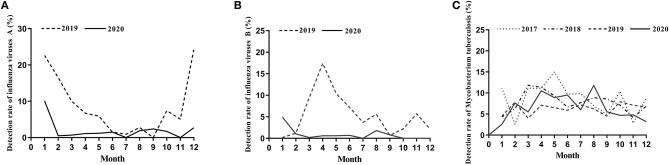
The detection rate of influenza viruses and Mycobacterium tuberculosis. **(A)** The detection rate of influenza viruses A from January to December during 2019–2020. **(B)** The detection rate of influenza viruses B from January to December during 2019–2020. **(C)** The detection rate of Mycobacterium tuberculosis from January to December during 2017–2020.

We found dramatic differences in the positive rate of influenza virus between 2019 and 2020. In 2019, the total positive rate of influenza virus A was 17.69%. There was a significant reduction in influenza A in 2020 compared to that of 2019, with only a 5.77% positive rate (Table 1). There were significant differences from January to May and October to December in the 2 years (Table 1 and Figure 1A). The influenza B epidemic in the 2 years was similar to that of influenza A, but slightly different. In 2019, the influenza B epidemic began in February and lasted for seven months until September, with the epidemic peak in April (Figure 1B). Nevertheless, during the same period in 2020, the testing data of influenza B showed the epidemic curve was flattened, among which the months with a statistical difference were from January to June, November and December except for February (Table 2 and Figure 1B). The total positive rate for influenza B was 3% in 2020 compared to the 5.15% positive rate in 2019 (Table 2).

**Table 1:** Progress of influenza A-like illness rate in 2019 and 2020.

**Month**	**2019**			**2020**			***P*-value**
	**No. of tested**	**Cumulative No. of ILI cases**	**ILI rate**	**No. of tested**	**Cumulative No. of ILI cases**	**ILI rate**	
1	862	195	22.60%	4,744	477	10.10%	<0.001
2	709	117	16.50%	1,518	8	0.50%	<0.001
3	841	84	10%	918	6	0.70%	<0.001
4	1,608	108	6.70%	464	5	1.10%	<0.001
5	776	46	5.90%	322	4	1.20%	0.001
6	264	4	1.50%	270	4	1.50%	0.974
7	109	1	0.90%	97	0	0	1
8	72	2	2.80%	53	1	1.90%	0.748
9	136	0	0.00%	123	3	2.40%	0.106
10	136	10	7.40%	115	2	1.70%	0.038
11	389	20	5.10%	127	0	0.00%	0.006
12	6932	1683	24.30%	182	5	2.70%	<0.001
Total	12,834	2,270	17.69%	8933	515	5.77%	<0.001

*ILI, influenza A-like illness*.

**Table 2:** Progress of influenza B-like illness rate in 2019 and 2020.

**Month**	**2019**			**2020**			***P*-value**
	**No. of tested**	**Cumulative No. of ILI cases**	**ILI rate**	**No. of tested**	**Cumulative No. of ILI cases**	**ILI rate**	
1	843	2	0.20%	4,672	228	14.90%	<0.001
2	691	8	1.20%	1,519	15	1%	0.715
3	831	78	9.40%	919	2	0.20%	<0.001
4	1,604	279	17.40%	464	3	0.60%	<0.001
5	778	81	10.40%	322	2	0.60%	<0.001
6	263	19	7.20%	270	2	0.70%	<0.001
7	107	4	3.70%	100	0	0.00%	0.122
8	72	4	5.60%	55	1	1.80%	0.283
9	135	1	0.70%	122	1	0.80%	0.943
10	134	3	2.20%	115	0	0.00%	0.251
11	388	22	5.70%	127	0	0.00%	0.004
12	6,737	147	2.20%	182	0	0.00%	0.034
Total	12,583	648	5.15%	8,558	254	3%	<0.001

*ILI, influenza B-like illness*.

### Epidemiology of Mycobacterium Tuberculosis in Patients From Henan Provincial People’s Hospital

The proportion of positive x-pert results for TB in 2017, 2018, 2019 and 2020 was 8.40, 7.69, 6.28, and 6.60%, respectively (Table 3). Figure 1C showed a slight downward trend before 2020. But contrary to the influenza A and B epidemic trend, data on detection of MTB showed a little higher positive rate in 2020 than that in 2019 (Table 3). Although the curve of MTB prevalence in 2020 was higher than in 2019, there was no statistical difference in the total positive rate (Table 3 and Figure 1C). Only in August, the incidence of Mycobacterium bacterium in 2020 was significantly higher than that in 2019(11.80 vs. 6.20%, *p* < 0.05) with statistical significance (Table 3).

**Table 3:** Progress of Mycobacterium tuberculosis detection rate from 2017 to 2020.

**Month**	**2017**			**2018**			**2019**			**2020**			***P*-value**
	**No. of tested**	**Cumulative No. of MTB cases**	**MTB rate**	**No. of tested**	**Cumulative No. of MTB cases**	**MTB rate**	**No. of tested**	**Cumulative No. of MTB cases**	**MTB rate**	**No. of tested**	**Cumulative No. of MTB cases**	**MTB rate**	
1	27	3	11.11%	185	8	4.32%	288	13	4.50%	115	3	2.60%	0.376
2	39	1	2.56%	128	8	6.25%	256	20	7.80%	66	5	7.60%	0.949
3	45	5	11.11%	186	22	11.83%	324	13	4.00%	91	5	5.50%	0.54
4	72	8	11.11%	176	20	11.36%	322	23	7.10%	153	16	10.50%	0.219
5	100	15	15.00%	215	20	9.30%	307	20	6.50%	168	15	8.90%	0.336
6	113	11	9.73%	166	11	6.63%	320	19	5.90%	201	19	9.50%	0.133
7	122	12	9.84%	208	16	7.69%	306	22	7.20%	184	11	6%	0.604
8	129	9	6.98%	237	21	8.86%	341	21	6.20%	178	21	11.80%	0.014
9	123	6	4.88%	221	19	8.60%	264	12	4.50%	165	9	5.50%	0.671
10	106	11	10.38%	238	17	7.14%	266	21	7.90%	172	8	4.70%	0.182
11	143	4	2.80%	264	11	4.17%	308	22	7.10%	228	11	4.80%	0.270
12	147	13	8.84%	248	17	6.85%	298	20	6.70%	278	9	3.20%	0.057
Total	1,166	98	8.40%	2,472	190	7.69%	3,600	226	6.28%	1,999	132	6.60%	0.633

*Statistical comparison between 2019 and 2020 was done using the Chi-square test*.

## Discussion

Our finding showed that public health interventions for COVID-19 in 2020 significantly reduced the spread of the influenza virus. As for the influenza virus, the decrease in the positive rate of specimens is an expected finding. Previous studies had shown that wearing a mask with frequent hand hygiene significantly reduced transmission of seasonal influenza virus in the community setting (7). Besides, there were also studies show that in addition to non-pharmaceutical interventions like mask-wearing and hand hygiene, other community hygienic measures such as social distancing and school closure resulted in a significant reduction of favorable rates of specimens detected of all circulating respiratory viruses including influenza viruses during the SARS outbreak in 2003 compared with preceding periods (8). However, the epidemic characteristics of influenza viruses vary from year to year. Multiple factors such as seasonal temperature, humidity, the number and characteristics of the virus in the circulating environment, immunity of the population, and the transmissibility of viruses will affect the extent of the influenza epidemic.

According to the epidemiological studies from 2014 to 2018, the influenza strains prevailing in Henan Province were seasonal influenza A virus H3 subtype strain, influenza A virus H1 subtype strain, and influenza B virus. In general, there are 2–3 serotypes co-circulate in each season and dominated by a single type (9). Henan Province belongs to the north of China. Data from the National influenza Center of China show that in the northern provinces of China, there was only one high peak incidence in 2020 (From January to March), while there was two high peak incidence during the 2019 influenza season (from January to March and from November and December). The epidemic strains were similar in the same peak incidence of 2019 and 2020 because of an alternation of the three prevailing strains involved in the epidemic peak. From the surveillance data of Henan Province in 2014–2018, the influenza-like illness (ILI) of children aged 0–14 accounted for 80.88% of all ILI. The influenza vaccination of children aged 0–14 started in July, reached a peak in October and ended in April and May of the next year. The amount of the influenza vaccination accounted for 38.03 and 61.97% of the total inoculation from July to October and from November to June of the next year (9). The COVID-19 epidemic began at the end of January 2020 and the period from February to June 2020 was when the country implemented strict non-pharmaceutical interventions, which may lead to a certain degree of reduction in the number of vaccinations administered. According to such results, the presumption that the vaccine’s effect on influenza protection is less than that of 2019 does not affect our conclusions, but rather reinforces our findings.

On the other hand, since Zhengzhou is a transport hub, the number of aviation and railway travelers is huge. At the beginning of the COVID-19 outbreak before implementing the intervention, sentinel hospital needs to screen the suspected patients who have lived in or traveled to Wuhan. Of the organized, experienced screening program, the influenza virus’s antigen detection is a necessary program to exclude the influenza-like illness. Possibly the reason why the number of people tested in January and February 2020 is more than the number of people tested at the same periods in 2019, resulting in the lower positive rate of influenza in January and February 2020. Reduced health-care-seeking behaviors during interventions implemented may also contribute to the results. Overall, however, the comprehensive public health interventions implemented, including mask-wearing and social distancing against COVID-19, seem to be the critical factor in the flattened epidemic curve of influenza in 2020 compared to the curve in 2019 (Figures 1B,C). Evidence supporting this finding that non-pharmaceutical interventions that reduced the COVID-19 transmission reduced influenza spread has been also verified in recently reported articles from other geographical regions or countries (10–12).

An interesting new aspect emerged with the discovery that the intervention doesn’t affect controlling MTB’s spread. The positive detection rate of MTB presents less variation in January, February, July, and September, but the positive rate in March, April, May, June, and August of 2020 was higher than that in the same period of 2019. Compared with last year, the positive rate was only slightly lower in November and December 2020, Overall, the MTB positive rate in 2020 was slightly higher than that in 2019 (Table 3 and Figure 1C). We have reasoned possible explanations for this phenomenon. First, during the implementation of protective measures, changes in behavior such as people stay indoors for a long time increased the risks of MTB infections. In previous studies of the seasonality of tuberculosis in India and South Africa, the authors suggested that increased tuberculosis disease transmission in winter may be due to increased indoor crowding in colder winter weather (13). Besides, since the transmission of tuberculosis was assumed to be an indoor event, it follows that transmission of MTB to a non-infected person is more likely if there was overcrowding and poor ventilation (14, 15). These results match well-with what we find. Previous study showed that social distancing during COVID-19 pandemic led to an increase in the number of dengue cases in Thailand (16). They reasoned that dengue spread at home rather than work addresses. Likewise, increased movement within residential neighborhoods may also increase the risk of MTB infection. Secondly, MTB can be carried in airborne particles called droplet nuclei that can be generated when persons who have pulmonary or laryngeal TB disease cough, sneeze, sing, or shout (17–19), so it is a pathogen of airborne transmission, what we called the aerosol transmission. Normal air currents can keep these approximately 1–5 um droplet nuclei containing infectious agents airborne for prolonged periods and spread them throughout a room or building, remaining infective over time and distance (18, 19). For airborne precautions, the CDC has recommended N95 or higher respirators to prevent airborne infectious agents (20). The N95 was also used as the gold standard for visitors to patients on airborne precautions (21). During the COVID-19 outbreak, the prevention measures included mask-wearing, but most people did not wear the N95 or higher respirators due to resource shortage or other factors, which may be the main reason for this result. However, as was the case with SARS, influenza is transmitted via the droplet (17, 18), which needs droplet precautions by wearing a surgical mask (20). Most people wear a surgical mask during the epidemic, which is sufficient for protection against influenza. These may be the causes for the difference between the intervention’s effects on the influenza virus and MTB. As described above, there was a statistical increase in the positive rate in August. During this month, a small peak of students returning to school and workers returning to work may be responsible for the increased MTB infection rate. However, after returning to work and school, the decrease of family crowding and community gathering may be the reason for the decrease of positive rate in November and December 2020.

The limitation of our research is that the specimens detected by x-pert are mostly from inpatients, which cannot reflect the detection rate of MTB in the whole population. Additionally, among non-pharmaceutical measures, including quarantine and isolation, social distancing, etc., we could not identify which measure was potentially the most effective and essential in suppressing influenza transmission. Further analysis will be needed to address these issues.

Though there is an effective vaccine against influenza, the risk for vaccine mismatch and loss of effectiveness also exists. Given that these precautions like social distancing, mask-wearing, and hand hygiene are practical, feasible, and acceptable, we might consider implementing them to control the influenza viral transmission during seasonal epidemics of influenza. Meanwhile, we should not ignore other airborne respiratory pathogens such as MTB when exerting our effort to protect against the respiratory viruses. On the contrary, different from influenza transmission, we need to pay more attention to them. In the future, with the gradual relaxation of quarantine, isolation and social distancing, and the occasional COVID-19 incident, we should wear the prescribed protective masks as far as possible if conditions permit.

## Data Availability Statement

The raw data supporting the conclusions of this article will be made available by the authors, without undue reservation.

## Ethics Statement

The studies involving human participants were reviewed and approved by ethical committee of Henan Provincial People’s Hospital. Written informed consent to participate in this study was provided by the participants’ legal guardian/next of kin.

## Author Contributions

LZ and GL conceived and designed the study. YG collected the data, analyzed the data, and wrote the initial draft. LZ analyzed the data and revised the manuscript. All authors contributed to the article and approved the submitted version.

## Conflict of Interest

The authors declare that the research was conducted in the absence of any commercial or financial relationships that could be construed as a potential conflict of interest.
